# Supplementary material for: Modulation of the Antibiotic Activity by the Mauritia flexuosa (Buriti) Fixed Oil against Methicillin-Resistant Staphylococcus Aureus (MRSA) and Other Multidrug-Resistant (MDR) Bacterial Strains
Source: Pathogens. 2018 Dec 10;7(4):98. doi: 10.3390/pathogens7040098 (PMC6313460; doi:10.3390/pathogens7040098)
Supplement: Supplementary file 1 [file pathogens-07-00098-s001.pdf]

LABORATÓRIO DE PESQUISAS DE PRODUTOS NATURAIS - LPPN
DEPARTAMENTO DE QUÍMICA BIOLÓGICA - DQB
CENTRO DE CIÊNCIAS BIOLÓGICAS E DA SAÚDE - CCBS
UNIVERSIDADE REGIONAL DO CARIRI - URCA

Informações - CG/EM

Analista: Fábio
Nome da Amostra: ErlanioBuriti_CHcl3
Volume de Injeção: 1 uL
Método: C:\GCMSsolution\Data\Project1\LPPN\Óleo Fixo.qgm
Tuning: C:\GCMSsolution\System\Tune1\07-02-17.qgt
Data: 7/2/2017

Chromatogram ErlanioBuriti_CHcl3

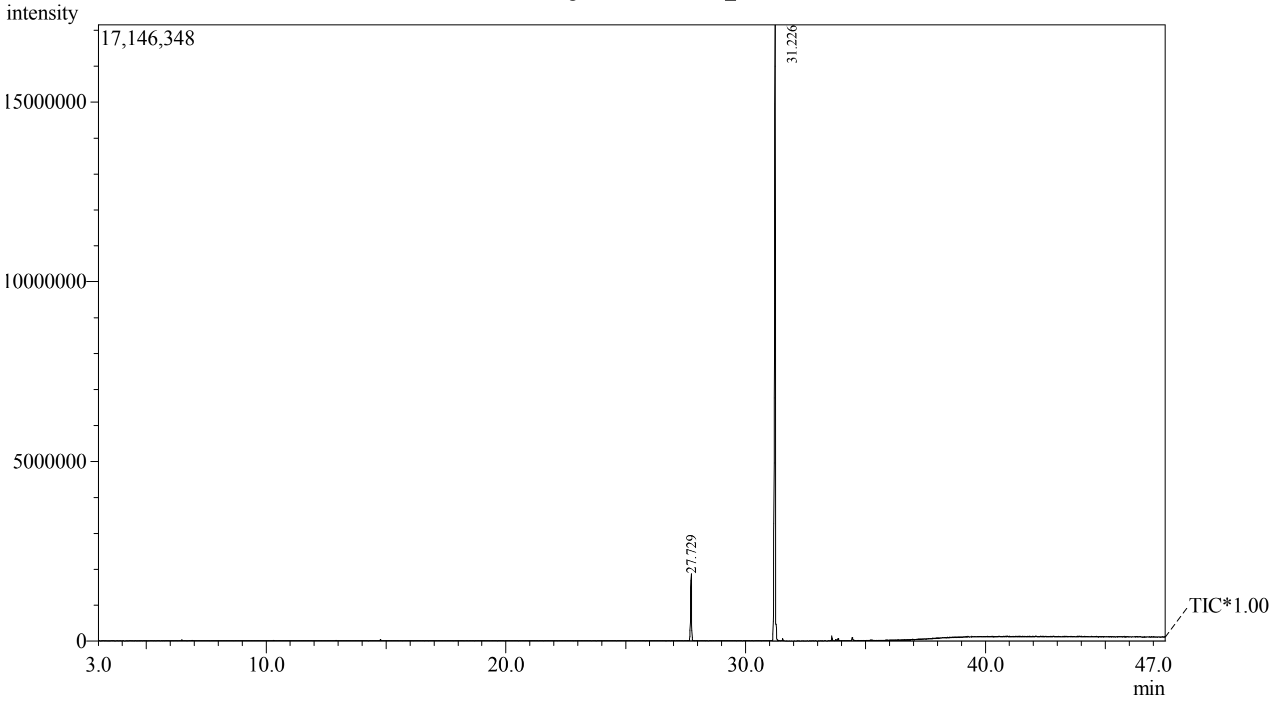

===== Analytical Line 1 =====

[GC-2010]
Column Oven Temp. :80.0 °C
Injection Temp. :280.00 °C
Injection Mode :Split
Pressure :65.2 kPa
Total Flow :54.0 mL/min
Column Flow :1.00 mL/min
Linear Velocity :36.8 cm/sec
Split Ratio :50.0
Oven Temp. Program

Rate	Temperature(°C)
-	80.0
5.00	180.0
10.00	300.0

If(3.0!=)Equilibrium Time :3.0 min

[GC Program]

===== Analytical Line 1 =====

[GCMS-QP2010]
[MS Table]
--Group 1 - Event 1--
Start Time :3.00min
End Time :47.50min
ACQ Mode :Scan
Event Time :0.50sec
Scan Speed :1111
Start m/z :43.00
End m/z :550.00

Sample Inlet Unit :GC

===== Analytical Line 1 =====

[GCMS-QP2010]
IonSourceTemp :200.00 °C
If(280.00!=)Interface Temp. :280.00 °C
(2.50!=)Solvent Cut Time :2.50 min

Peak Report TIC

Peak#	R.Time	Area	Area%	Height	A/H	Mark	Name	Base m/z
1	27.729	5582888	10.19	1865235	2.99			74.00
2	31.226	49230099	89.81	17138516	2.87	V		55.05
		54812987	100.00	19003751				

Library

<< Target >>
Line#:1 R.Time:27.725(Scan#:2968) MassPeaks:92
RawMode:Averaged 27.717-27.733(2967-2969) BasePeak:74.00(394206)
BG Mode:Calc. from Peak Group 1 - Event 1

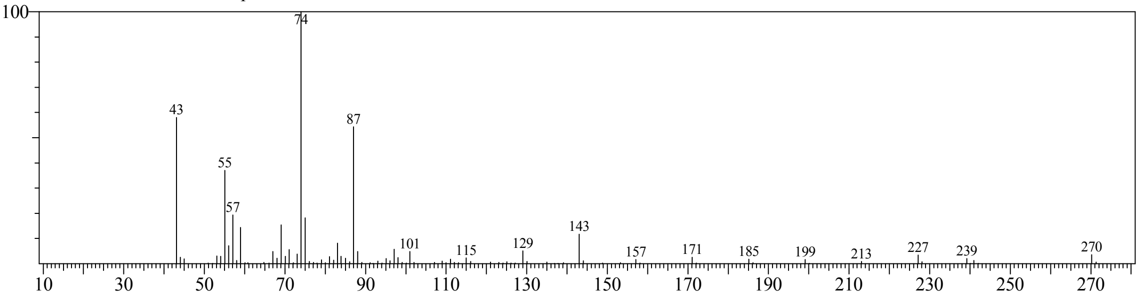

Hit#:1 Entry:90720 Library:NIST08.LIB
SI:95 Formula:C17H34O2 CAS:5129-60-2 MolWeight:270 RetIndex:1814
CompName:Pentadecanoic acid, 14-methyl-, methyl ester $$ Methyl 14-methylpentadecanoate # $$

Hit#:2 Entry:22754 Library:NIST08s.LIB
SI:94 Formula:C17H34O2 CAS:112-39-0 MolWeight:270 RetIndex:1878
CompName:Hexadecanoic acid, methyl ester $$ Palmitic acid, methyl ester $$ n-Hexadecanoic acid methyl ester $$ Metholene 2216 $$ Methyl hexadecano[illegible]

Hit#:3 Entry:100813 Library:NIST08.LIB
SI:93 Formula:C18H36O2 CAS:6929-04-0 MolWeight:284 RetIndex:1914
CompName:Hexadecanoic acid, 15-methyl-, methyl ester $$ Methyl isoheptadecanoate $$ Methyl 15-methylhexadecanoate $$

Hit#:4 Entry:70951 Library:NIST08.LIB
SI:92 Formula:C15H30O2 CAS:5129-58-8 MolWeight:242 RetIndex:1615
CompName:Tridecanoic acid, 12-methyl-, methyl ester $$ Methyl isomyristate $$ Methyl 12-methyltridecanoate $$

Hit#:5 Entry:19697 Library:NIST08s.LIB
SI:92 Formula:C14H28O2 CAS:1731-88-0 MolWeight:228 RetIndex:1580
CompName:Tridecanoic acid, methyl ester $$ Methyl tridecanoate $$ n-Tridecanoic acid methyl ester $$ Methyl ester of tridecanoic acid $$

<< Target >>
Line#:2 R.Time:31.225(Scan#:3388) MassPeaks:193
RawMode:Averaged 31.217-31.233(3387-3389) BasePeak:55.05(1702177)
BG Mode:Calc. from Peak Group 1 - Event 1

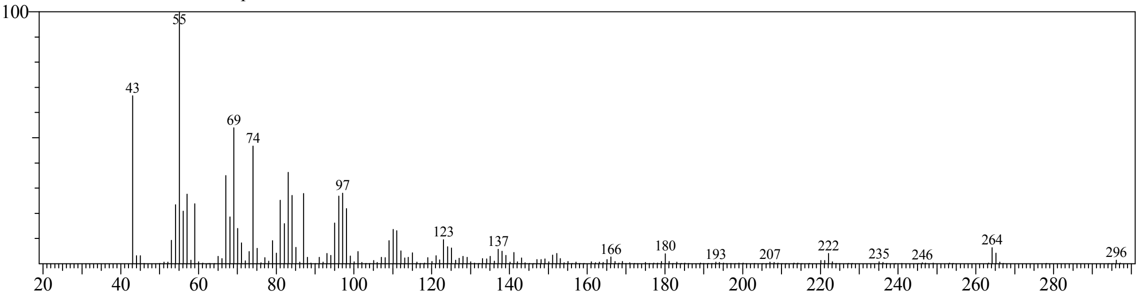

Hit#:1 Entry:24158 Library:NIST08s.LIB
SI:95 Formula:C19H36O2 CAS:112-62-9 MolWeight:296 RetIndex:2085
CompName:9-Octadecenoic acid (Z)-, methyl ester $$ Oleic acid, methyl ester $$ Emery oleic acid ester 2301 $$ Methyl cis-9-octadecenoate $$ Methyl ole[illegible]

Hit#:2 Entry:109248 Library:NIST08.LIB
SI:94 Formula:C19H36O2 CAS:13481-95-3 MolWeight:296 RetIndex:2085
CompName:10-Octadecenoic acid, methyl ester $$ Methyl (10E)-10-octadecenoate # $$

Hit#:3 Entry:24156 Library:NIST08s.LIB
SI:94 Formula:C19H36O2 CAS:1937-62-8 MolWeight:296 RetIndex:2085
CompName:9-Octadecenoic acid, methyl ester, (E)- $$ Elaidic acid, methyl ester $$ Methyl elaidate $$ Methyl trans-9-octadecenoate $$ (E)-9-Octadecenoi[illegible]

Hit#:4 Entry:109241 Library:NIST08.LIB
SI:93 Formula:C19H36O2 CAS:52380-33-3 MolWeight:296 RetIndex:2085
CompName:11-Octadecenoic acid, methyl ester $$ Methyl 11-octadecenoate $$ Octadec-11-enoic acid, methyl ester $$ Methyl (11E)-11-octadecenoate # $[illegible]

Hit#:5 Entry:109250 Library:NIST08.LIB
SI:93 Formula:C19H36O2 CAS:56554-47-3 MolWeight:296 RetIndex:2085
CompName:13-Octadecenoic acid, methyl ester $$ Methyl (13E)-13-octadecenoate # $$
